# The role and mechanism of NLRP3 inflammasome-mediated astrocyte activation in dehydrocorydaline against CUMS-induced depression

**DOI:** 10.3389/fphar.2022.1008249

**Published:** 2022-11-23

**Authors:** Yu Fang, Hong Guo, Qiannan Wang, Congcong Liu, Shuyi Ge, Bohua Yan

**Affiliations:** ^1^ Department of Neurology, Hospital of Chengdu University of Traditional Chinese Medicine, Chengdu, China; ^2^ Department of Rehabilitation, Hospital of Chengdu University of Traditional Chinese Medicine, Chengdu, China; ^3^ Clinical Medicine, Graduate School of Chengdu University of Traditional Chinese Medicine, Chengdu, China; ^4^ Department of GCP, Hospital of Chengdu University of Traditional Chinese Medicine, Chengdu, China

**Keywords:** depression, CUMS, NLRP3 inflammasome, astrocyte activation, dehydrocorydaline

## Abstract

**Background:** Depression is a common and potentially life-threatening mental illness, and currently, there is a lack of effective treatment. It has been reported that dehydrocorydaline (DHC) can inhibit monoamine transporter uptake in depressed CUMS mice, but more possible mechanisms of action remain to be further studied.

**Methods:** C57BL/6 mice were exposed to chronic unpredictable mild stress (CUMS) for five consecutive weeks. The mice were administrated with dehydrocorydaline or fluoxetine (FLU) for four consecutive weeks. Behavioral tests including sucrose preference test (SPT), tail suspension test (TST), and forced swimming test (FST) were applied. In parallel, hematoxylin and eosin (H&E) staining and Nissl staining were used to explore the effect of DHC on pathological changes in the hippocampus. The concentrations of depression-related factors (5-HT and DA) and inflammatory factors (TNF-α, IL-6, and IL-1β) in the hippocampus and serum were assessed by ELISA assay. NLRP3 inflammasome pathway-related proteins (NLRP3, IL-18, IL-1 IL-1α, and caspase-1) were detected by western blot. The activation of microglia and astrocytes was subjected to immunofluorescent staining. Additionally, microglia were treated with DHC (100 mg/L) for 24 h following incubation with 100 ng/ml LPS for 12 h. ov-NC or ov-NLRP3 plasmid was transfected into microglia 6 h before LPS induction for exploring the effect of NLRP3 overexpression on DHC-inhibited microglia activation. Then, conditioned media of microglia were collected from each group, followed by intervention of astrocytes for 24 h to explore the effect of NLRP3 overexpression of microglia on astrocyte activation.

**Results:** *In vivo* administration of DHC was found to ameliorate depressive-like behaviors and attenuate neuron damage of CUMS mice. DHC increased neurotransmitter concentration, reduced the proinflammatory factor levels, attenuated NLRP3 inflammasome activation, and decreased A1 astrocyte and microglia activation in the hippocampus of CUMS mice. Furthermore, *in vivo* results showed that activated microglia induced activation of A1 astrocytes but not A2 astrocytes.

**Conclusion:** Taken together, we provided evidence that DHC exhibited antidepressive effects on CUMS mice possibly *via* NLRP3 inflammasome-mediated astrocyte activation.

## Introduction

Depression is a category of common psychiatric mood disorders (ÇALIŞKAN and DOST, 2020; McCarron et al., 2021; Poulida and Makra, 2021). It has been reported that approximately 17% of people experience depression at least once in their lifetime (Andrade et al., 2003). Several mutual factors, such as psychological, epigenetic, and social factors, can contribute to depression (Gaiteri et al., 2014). Prominently, chronic stress and physical pain can evoke depression and influence progression and severity (Lee et al., 2009; Agüera-Ortiz et al., 2011). Therefore, depression cases are always heterogeneous due to neurobiological changes, clinical progression, genetic influences, and therapy responses to antidepressants (van Loo et al., 2012; de Vos et al., 2015). Patients with depression show social withdrawal, anxiety, depressed mood, apathy, disturbed sleep, memory deficits, psychomotor retardation, and alteration in food consumption (McCarron et al., 2021). It has been demonstrated that multiple methods are applied in the treatment of depression, such as antidepressants, psychological interventions, psychotherapies, transcranial direct current stimulation, and their combination (Zhou et al., 2017; Zhou et al., 2020; Wang et al., 2021a). Nevertheless, the efficacy and safety of these approaches are still undetermined. Thus, continual exploration for effective and safe antidepressants is obligatory and important.

*Corydalis yanhusuo* (Y.H.Chou and Chun C.Hsu) W.T. Wang ex Z.Y. Su and C.Y. Wu (*Papaveraceae*; *Corydalis rhizoma*), with slight qi and a bitter taste, has the functions of promoting blood circulation, invigorating qi, and analgesia, which is often used in clinical treatment of coronary heart disease, arrhythmia, gastric ulcer, and other conditions (Tian et al., 2020). The main active components of *Corydalis yanhusuo* (Y.H.Chou and Chun C.Hsu) W.T. Wang ex Z.Y. Su and C.Y. Wu (*Papaveraceae*; *Corydalis rhizoma*) are alkaloids, which have various pharmacological activities, such as anti-inflammatory, analgesic, antitumor, antibacterial and antiviral, and cardiovascular activities (Tian et al., 2020). Xu (2015) report that the content of the alkaloid dehydrocorydaline in the tubers and leaves of *Corydalis yanhusuo* (Y.H.Chou and Chun C.Hsu) W.T. Wang ex Z.Y. Su and C.Y. Wu (*Papaveraceae*; *Corydalis rhizoma*) is the highest. It has been known that dehydrocorydaline has good curative effects on coronary heart disease, myocardial infarction, and gastric ulcer (Shoji et al., 1974; Guan et al., 2017; Chen et al., 2020). Dehydrocorydaline may suppress platelet aggregation *via* a mechanism involving the ADP receptors P2Y1 and P2Y12, inhibit antibody-mediated allergic reactions, and influence cell-mediated allergic reactions (Tan et al., 2019). Furthermore, dehydrocorydaline may produce an anti-inflammatory effect through a decreased expression of caspase 6 (CASP6), TNF-α, IL-1β, and IL-6 proteins in the spinal cord (Yin et al., 2016). Moreover, Jin et al. (2019) reveal that dehydrocorydaline induces an antidepressant-like effect in a chronic unpredictable mild stress mouse model *via* inhibiting uptake-2 monoamine transporters. However, the underlying mechanism involving in the antidepressant-like effect of dehydrocorydaline remains defective.

Thus, in this study, the role and potential mechanism of dehydrocorydaline in depression were explored in mice subjected to chronic unpredictable mild stress (CUMS). We hope our outcomes can lay a theoretical basis for the therapy of depression and other associated disorders.

## Materials and methods

### Animals

A total of 60 C57BL/6 male mice weighing 16–20 g (5–6 weeks old) were obtained from Chengdu Dossy Experimental Animals Co., Ltd. The mice were housed in polycarbonate cages (10 animals per cage) for 1 week under standard conditions, including a 24 ± 1 °C temperature control, a 12 h light/12 h dark cycle, a humidified atmosphere (50 ± 10%), and free access to food and water. All experiments were performed in accordance with the guidelines of the China Council on Animal Care and were approved by the Ethical Committee of the Chengdu University of Traditional Chinese Medicine (Ethical code: 2022DL-014. Chengdu, China).

### Groups and drug administration

The mice were randomly assigned to six groups (*n* = 10) as follows: control group, CUMS group, low-dose dehydrocorydaline group (L-DHC, 50 mg/kg), media-dose dehydrocorydaline group (M-DHC, 100 mg/kg), high-dose dehydrocorydaline group (H-DHC, 200 mg/kg), and fluoxetine group (FLU, positive control, 20 mg/kg). Following 5 weeks of CUMS, mice in the DHC group and the FLU group were intragastrated with associated doses of dehydrocorydaline and fluoxetine once a day for 4 weeks. Mice in the control group and CUMS group were intragastrated with an equal volume of saline. The gavage volume was 0.1 ml/10 g (weight). The DHC dosages selected in this study were according to Li et al. (Jin et al., 2019). Behavioral tests were performed on all of the mice after the last day of administration.

A depression model was induced by two random stressors per day. The mild stressors included tilting the cage at 45° for 24 h, food deprivation for 24 h, water deprivation for 24 h, wet bedding for 24 h, swimming in 4 °C water for 5 min, shaking at 120 rpm for 30 min, restraint in a 50 ml tube for 6 h, and illumination throughout the night (overnight illumination occurred twice per week) (Wang et al., 2021b). The control mice were group-housed under normal conditions.

### Sucrose preference test

As described in the previous description, sucrose preference trials were conducted in cages filled with two standard drinking bottles filled with 2% sucrose solution (w/v) (Fernandes and Gupta, 2019). After consuming sucrose solution for 24 h, mice were given free access to drinking water for 3 h, after which two bottles were replaced with 2.5% sucrose solution and tap water. In order to avoid side preferences, the two drinking bottles were switched halfway through the test. The sucrose preference was observed.

### Tail suspension test

A tail suspension test determines the degree of desperation and helplessness in mice (Li et al., 2019). We suspended the mice 35 cm above the floor for 6 min with adhesive tape (1–2 cm from the beginning of the tail) and measured their immobility over the last 4 min.

### Forced swimming test

To determine depression-like behavior, a forced swimming test was conducted (Mohseni et al., 2017). A transparent cylinder with a 25 cm diameter and a 60 cm height was filled to a depth of about 30 cm with water (25 ± 2°C). Prior to the trail, mice were pretested for 15 min and then tested for 5 min the next day. The immobility time was documented when mice remained motionless in an upright location or floated with only enough movement requisite to maintain their head above the water level.

### Hematoxylin and eosin staining

Hippocampus tissues were separated from the brain tissues and immobilized in 4% paraformaldehyde for 48 h. Then, slices of the hippocampus tissues (5 µm) were cut continuously and stained with H&E after being embedded, dehydrated, and embedded in calcium phosphate solution. Images of stained slices were taken with a digital trinocular camera microscope (CX23, Olympus, Tokyo, Japan).

### Nissl staining

Nissl staining was used to determine the damage to neurons in the hippocampus.

Briefly, the dewaxed hippocampus slices were stained with the Nissl stain solution (Methyl Violet Method) (G1432, Solarbio) following the working instructions. The slices were imaged under a digital trinocular camera microscope after being dehydrated, hyalinized, and mounted (CX23, Olympus).

### ELISA assay

Immediately after an intraperitoneal injection of 3% pentobarbital sodium (50 mg/kg) for anesthesia, brain tissues were removed, placed on an ice tray to isolate hippocampus tissues, and weighed. As a next step, the tissues were shredded using an ophthalmic scissors and mixed with normal saline (1:10) to produce 10% brain tissue homogenate. The homogenate was centrifuged at 4 °C for 10 min to recover the supernatant. In addition, serum was collected for subsequent use. 5-HT, DA, TNF-α, IL-6, and IL-1β were detected by the colorimetry method using a microplate reader according to the kit instructions.

An ultrasonic cell disrupter was used to disrupt each group’s cells at 4°C, and the lysates were centrifuged at 1,000 r/min and 4 °C for 10 min. A total of 100 μl of the supernatant was used to determine the OD values using a microplate reader according to the instructions of the TNF-α, IL-1α, and PGE2 kit.

### Western blot

Total proteins from brain tissues with the hippocampus were extracted with a Total Protein Extraction Kit (BC3711, Solarbio) and quantified by the BCA protein quantification kit (ab102536, Abcam, Cambridge, UK) in line with the operation manual. 10 %SDS-PAGE was used to dissolve protein samples and electrically transfer them to PVDF membranes. For the block, the membrane was treated for 1 h at room temperature with 3% bovine serum albumin (BSA) and then primed overnight at 4 °C with primary antibodies. As a next step, the membrane was washed three times and hatched for 1 hour at 37 °C with appropriate secondary antibodies. An ECL chemiluminescence kit (WBULS0500; EMD Millipore) was used to visualize the bands. The primary antibodies were NLRP3 (1:1000), Iba-1 (1:10,000), IL-18 (1:500), IL-1β (1:1000), and caspase1 (1:1000), and the second antibody was Goat anti-rabbit IgG H&L (HRP) (1:20,000), all of which were purchased from Abcam.

### Immunofluorescence assay

After dewaxing, the paraffin sections were subjected to antigen retrieval with citrate buffer (pH = 6). Then, sections were rinsed with phosphate buffer saline (PBS, Beyotime, Shanghai, China) thrice and blocked with goat serum (SP9002, ZSGB-BIO, Beijing, China) for 20 min at the room temperature. Next, sections were incubated with CD68, Iba-1, C3, and the GFAP antibody overnight at 4°C. Subsequently, sections were washed with PBS three times and incubated with FITC conjugated Goat anti-rabbit IgG (H + L) (1:100, GB22303, Servicebio, Wuhan, China) or Cy3-conjugated Goat anti-rabbit IgG (H + L) (1:100, GB21303, Servicebio) at 37 °C for 30 min. The nuclei were stained with DAPI (ZLI-9557, ZSGB-BIO) for 10 min at room temperature. Images were captured with a confocal microscope (LSM700; Zeiss, Oberkochen, Germany).

### Cell lines and cell culture

Mice microglial cell lines (BV2; CL-0493) and mouse astrocytes (CP-M157) were purchased from Procell Life Science & Technology Co., Ltd. BV2 cells were cultured in a BV2 cell culture medium (MEM+10% FBS+1% P/S; CM-0493; Procell). Astrocytes were cultured in a mouse astrocyte complete culture medium (CM-M157; Procell). All cells were incubated at 37 °C in 5% CO_2_.

### Cell transfection and treatment

The pcDNA-NLRP3 (ov-NLRP3) and pcDNA (ov-NC) vectors were designed and synthesized by Shanghai GenePharma Technology Co., Ltd. All of the *in vitro* transfection was performed using Zeta Life Advanced DNA transfection reagent (Zeta Life, CA, United States) according to the manufacturer’s protocol after BV2 cells were grown to 70–80% confluence.

BV2 cells were incubated with DHC (100 mg/L) and 100 ng/ml LPS for 12 h. ov-NC or ov-NLRP3 plasmid was transfected into BV2 cells 6 h before LPS induction. Conditioned media of BV2 cells were collected from each group *via* centrifugation at 4 °C with 12,000 g for 10 min, followed by intervention of astrocytes for 24 h.

### Statistical analysis

All results were exhibited as the mean ± standard deviation (SD). Data were tested by the Student’s t-test for two groups or one-way analysis of variance and Duncan’s test for more than two groups using the SPSS 22.0 package (SPSS Inc. Chicago, IL, United States), followed by the *Post Hoc* Bonferroni test. The differences were regarded as statistically significant when *p* < 0.05.

## Results

### Dehydrocorydaline ameliorated depressive-like behaviors and decreased body weight in chronic unpredictable mild stress-induced depression in mice

**FIGURE 1:**
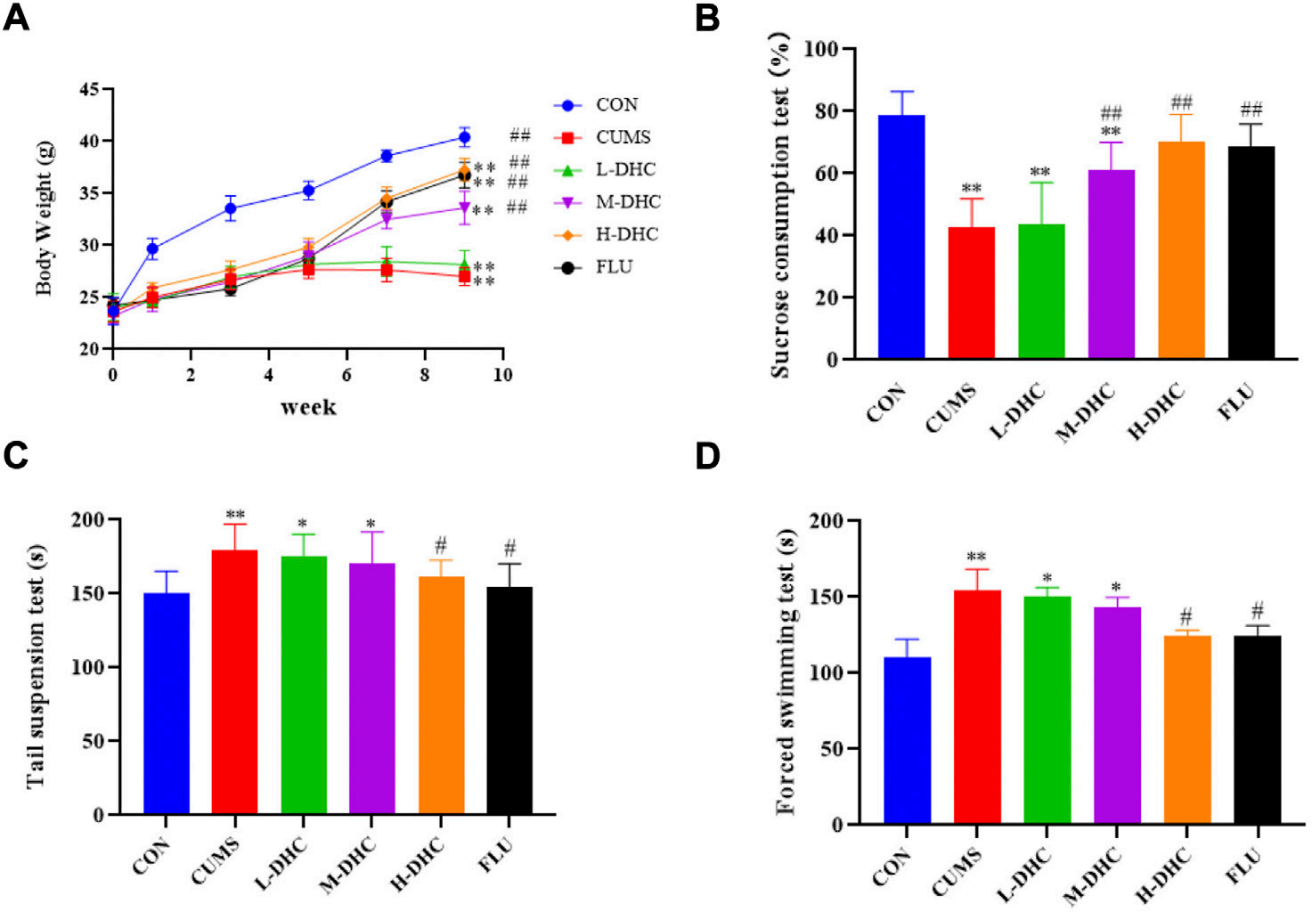
Effects of DHC or fluoxetine on body weight and desperate behavior of CUMS-induced depression in mice. **(A)** Body weight, **(B)** sucrose preference test (SPT), **(C)** tail suspension test (TST), and **(D)** forced swimming test (FST). ^*^ *p* < 0.05 and ^**^ *p* < 0.01, compared with the control (CON) group; ^#^ *p* < 0.05 and ^##^ *p* < 0.01, compared with the CUMS group.

Following administration of DHC for 4 weeks, all mice underwent body weight and behavioral tests including the sucrose preference test (SPT), forced swimming test (FST), and tail suspension test (TST). As shown in Figure 1A, the DHC for four weeks significantly reduced the weight loss induced by CUMS at 8 weeks. Also, the treatment of DHC significantly reduced depressive-like behaviors of CUMS-induced depression in mice, which showed that compared with the model group, sucrose intake of mice in the DHC treatment group was significantly increased, and immobile time in FST and TST was significantly reduced (Figures 1B–D). These results indicated the antidepressive effects of DHC in CUMS-induced depression in mice.

### Dehydrocorydaline attenuated neuron damage in chronic unpredictable mild stress-induced depression in mice

**FIGURE 2:**
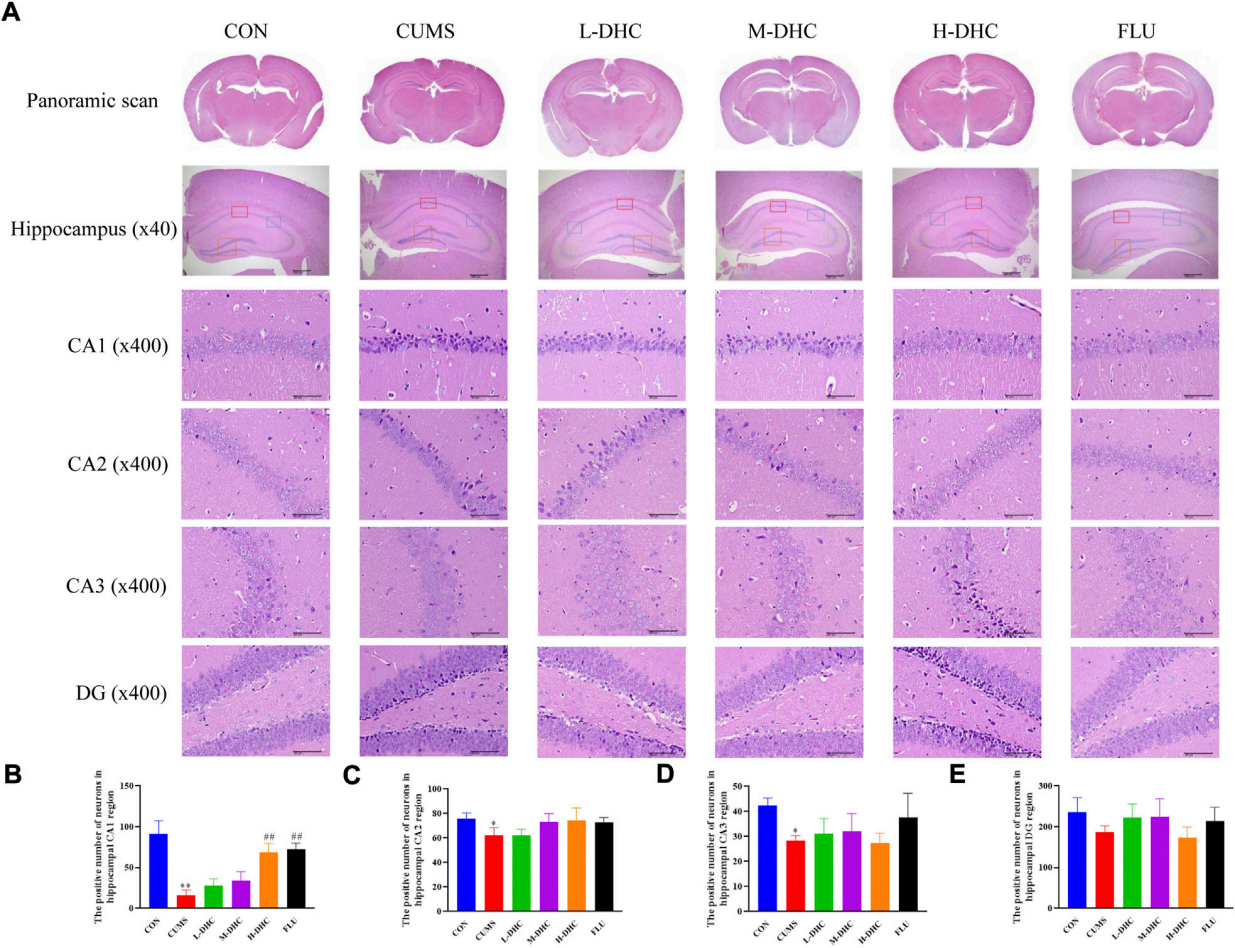
Effects of DHC or fluoxetine on the morphological changes of the hippocampal region in CUMS-induced depression in mice. **(A)** Representative images of hematoxylin-eosin (H&E) staining in the hippocampal region of brain tissue. Previously mentioned is a panoramic scan of brain tissue after H&E staining; the following image is a HE staining of the CA1, CA2, CA3, and DG regions of the hippocampus, ×400 magnification (scale bar 50 μm). The red box represents the CA1 region, the blue box represents the CA2 region, the green box represents the CA3 region, and the orange represents the DG region; **(B)** the positive number of neurons in the hippocampal CA1 region of mice in each group; **(C)** the positive number of neurons in the hippocampal CA2 region of mice in each group; **(D)** the positive number of neurons in the hippocampal CA3 region of mice in each group; and **(E)** the positive number of neurons in the hippocampal DG region of mice in each group. ^*^ *p* < 0.05 and ^**^ *p* < 0.01, compared with the control (CON) group; ^##^ *p* < 0.01, compared with the CUMS group.

**FIGURE 3:**
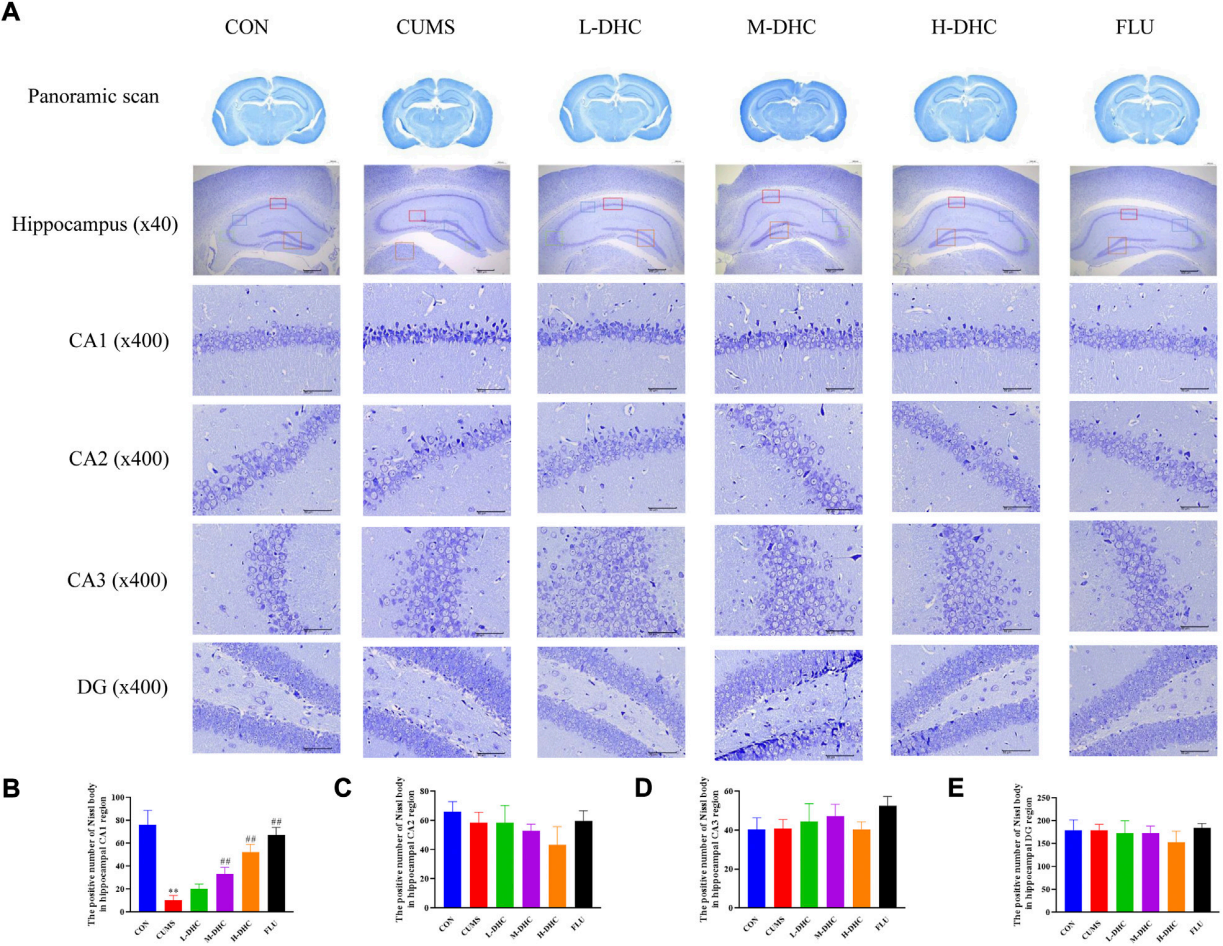
Effects of DHC or fluoxetine on the damage of hippocampus neurons in CUMS-induced depression in mice. **(A)** Representative images of Nissl staining of the hippocampal region. Previously mentioned is a panoramic scan of brain tissue after Nissl staining; the following image is Nissl staining of the CA1, CA2, CA3, and DG regions of the hippocampus, ×400 magnification (scale bar 50 μm). The red box represents the CA1 region, the blue box represents the CA2 region, the green box represents the CA3 region, and the orange represents the DG region; **(B)** the positive number of Nissl body in the hippocampal CA1 region of mice in each group; **(C)** the positive number of Nissl body in the hippocampal CA2 region of mice in each group; **(D)** the positive number of Nissl body in the hippocampal CA3 region of mice in each group; **(E)** the positive number of Nissl body in the hippocampal DG region of mice in each group. ^**^ *p* < 0.01, compared with the control (CON) group; ^##^ *p* < 0.01, compared with the CUMS group.

Morphological changes in the hippocampal region were measured with H&E staining (Figure 2). A large number of neuron necrosis, cytoplasmic, nuclear pyknosis, and microglial proliferation were observed in the hippocampus of CUMS mice, while the high dose DHC and fluoxetine could reverse these pathological changes. In Nissl staining, the CUMS challenge resulted in significant neurodegeneration, with a reduced proportion of viable neurons, which could be markedly ameliorated following DHC or fluoxetine administration (Figure 3). These results suggested that DHC had a protective effect on CUMS-induced hippocampal neurons in CUMS-induced depression in mice.

### Dehydrocorydaline increased the neurotransmitter concentration and reduced the proinflammatory factor levels in chronic unpredictable mild stress-induced depression in mice

**FIGURE 4:**
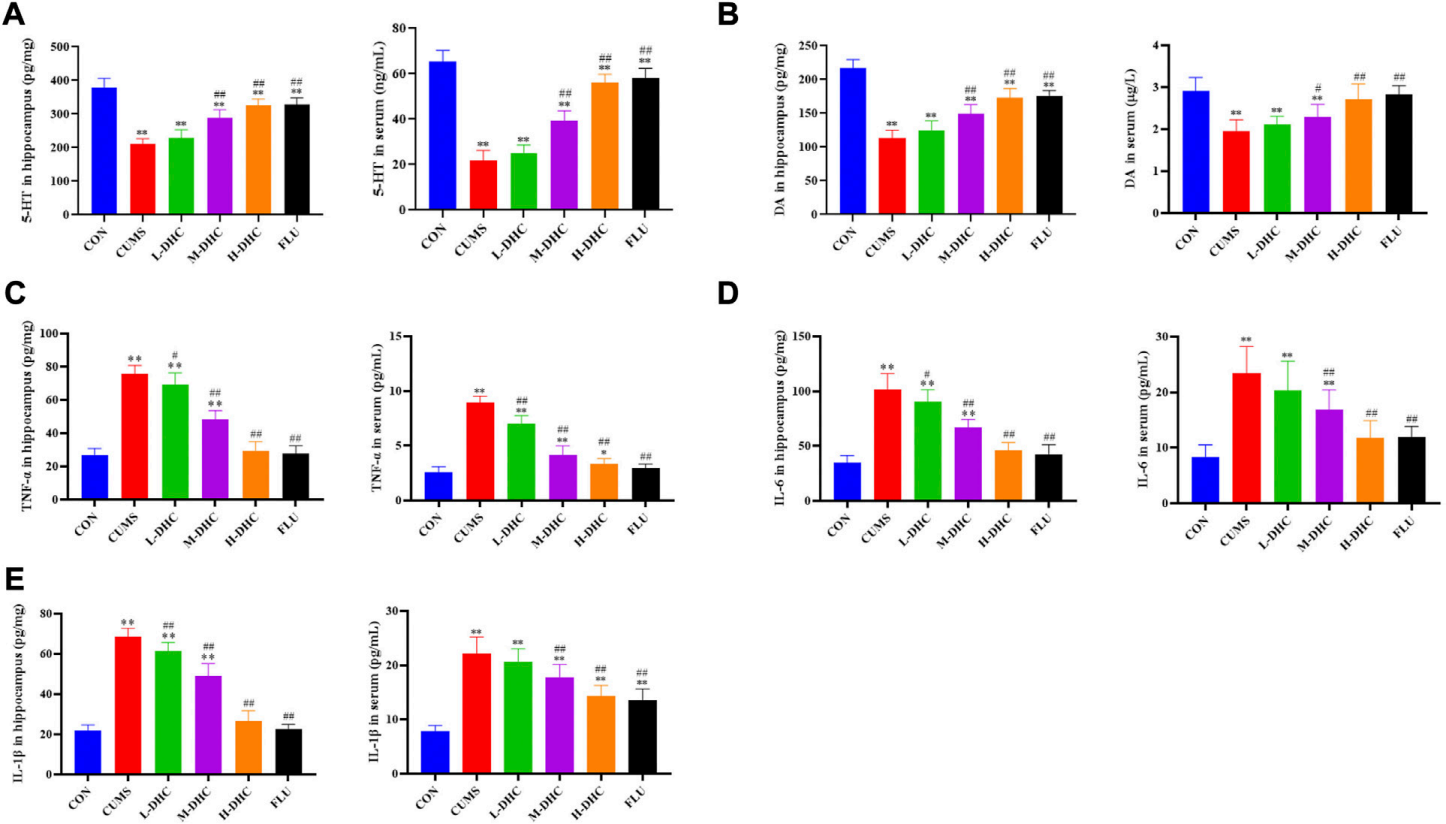
Effects of DHC or fluoxetine on 5-HT, DA, and proinflammatory factor levels in CUMS-induced depression in mice. **(A)** 5-HT level was determined in serum and hippocampus, **(B)** DA level was determined in serum and hippocampus, **(C)** TNF-α level was determined in serum and hippocampus, **(D)** IL-6 level was determined in serum and hippocampus, and **(E)** IL-1β level was determined in serum and hippocampus. ^*^ *p* < 0.05 and ^**^ *p* < 0.01, compared with the control (CON) group; ^#^ *p* < 0.05 and ^##^ *p* < 0.01, compared with the CUMS group.

Following DHC or fluoxetine treatment for four weeks, the concentrations of 5-HT and DA in the serum and hippocampus were increased significantly compared with those of the CUMS group (Figures 4A,B). The levels of TNF-α, IL-6, and IL-1β in the serum and hippocampus were markedly downregulated after DHC or fluoxetine treatment compared with those of the CUMS group (Figures 4C–E). All of the aforementioned data indicated that DHC increased neurotransmitter concentration and attenuated neuroinflammation of CUMS mice.

### Dehydrocorydaline attenuated NLRP3 inflammasome activation in the hippocampus of chronic unpredictable mild stress-induced depression in mice

**FIGURE 5:**
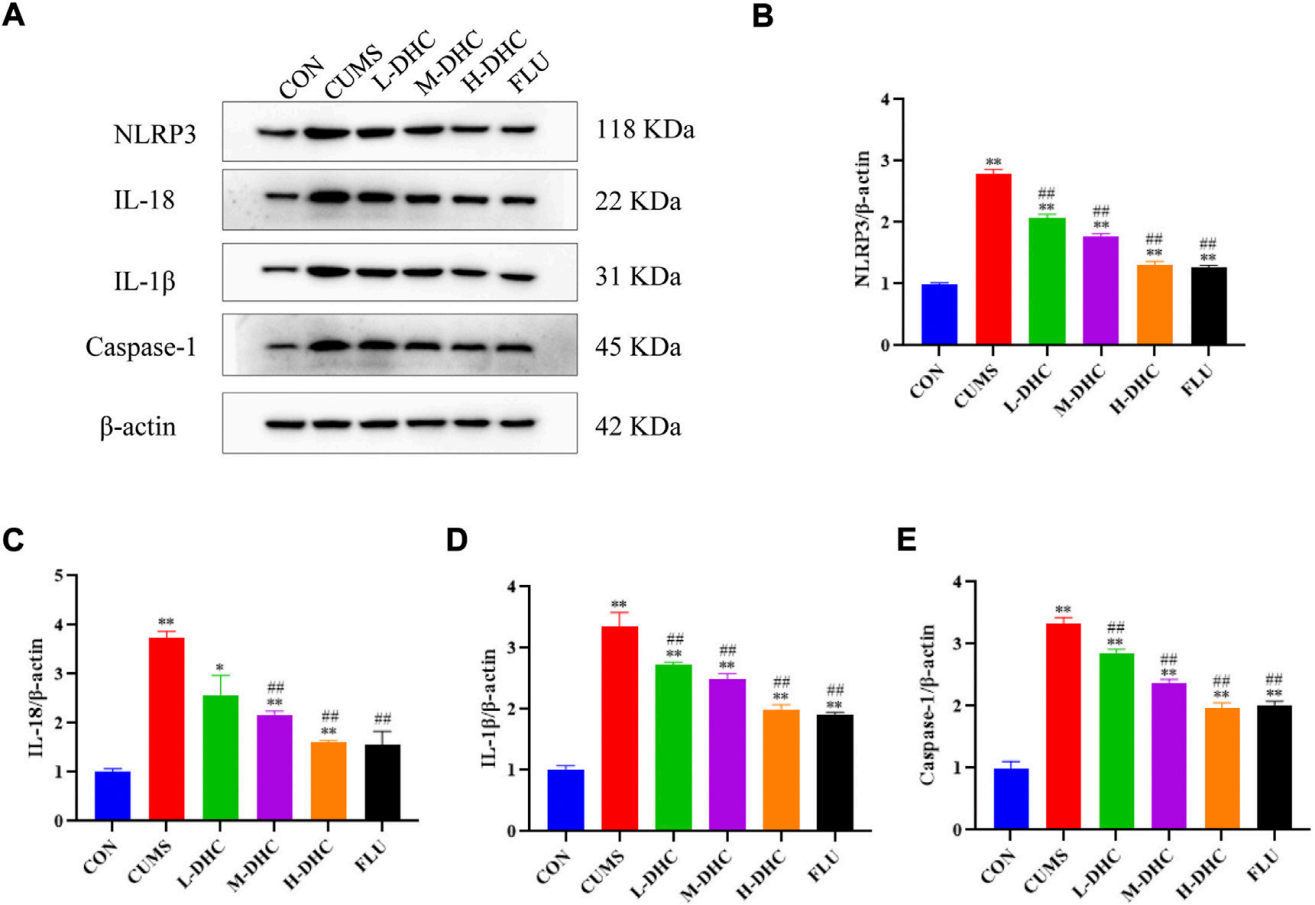
Effects of DHC or fluoxetine on NLRP3 inflammasome activation in CUMS-induced depression in mice. **(A)** Image of NLRP3 inflammasome-associated protein in the hippocampus. β-actin was used as an internal reference, **(B)** the expression level of NLRP3 protein, **(C)** the expression level of IL-18 protein, **(D)** the expression level of IL-1β protein, and **(E)** the expression level of caspase-1 protein. ^*^ *p* < 0.05 and ^**^ *p* < 0.01, compared with the control (CON) group; ^#^ *p* < 0.05 and ^##^ *p* < 0.01, compared with the CUMS group.

To further investigate the mechanism of DHC’s anti-inflammatory effect on CUMS mice, the expression of NLRP3, IL-18, IL-1β, and caspase-1 was detected. NLRP3 inflammasome is an inflammatory signaling molecular complex that plays a critical role in depression. As shown in Figure 5, the results showed that DHC significantly downregulated the expression of NLRP3 inflammasome associated proteins, including IL-18, IL-1β, and caspase-1, in the hippocampus of CUMS mice compared with the CUMS group. These results suggested that DHC might suppress the release of IL-18 and IL-1β by inhibiting NLRP3 expression.

### Dehydrocorydaline decreased A1 astrocyte and microglia activation in the hippocampus of chronic unpredictable mild stress-induced depression in mice

**FIGURE 6:**
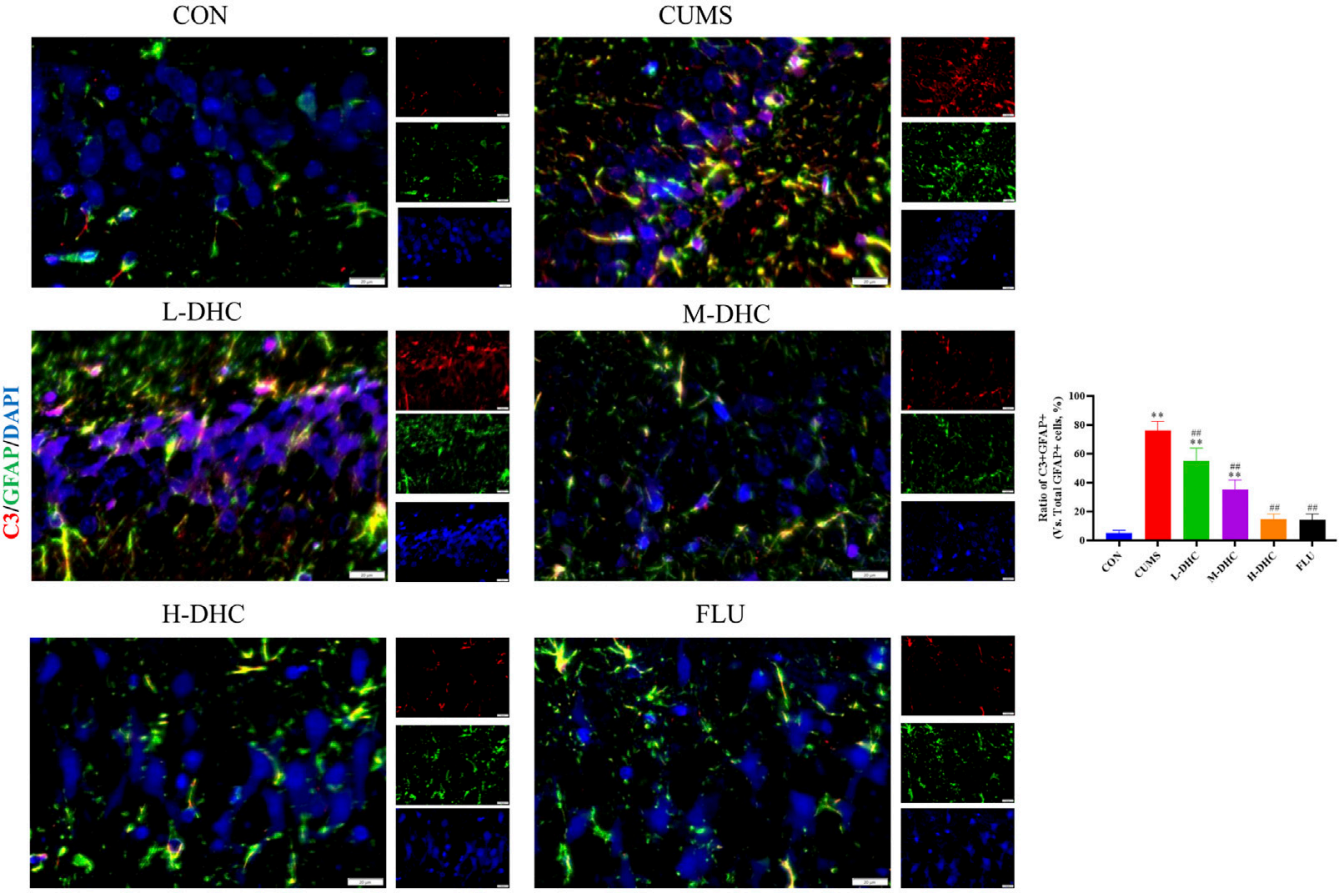
Effects of DHC or fluoxetine on A1 astrocyte activation in CUMS-induced depression in mice. Red fluorescence represented C3 positive cells and green fluorescence represented GFAP positive cells. ^*^ *p* < 0.05 and ^**^ *p* < 0.01, compared with the control (CON) group; ^#^ *p* < 0.05 and ^##^ *p* < 0.01, compared with the CUMS group.

**FIGURE 7:**
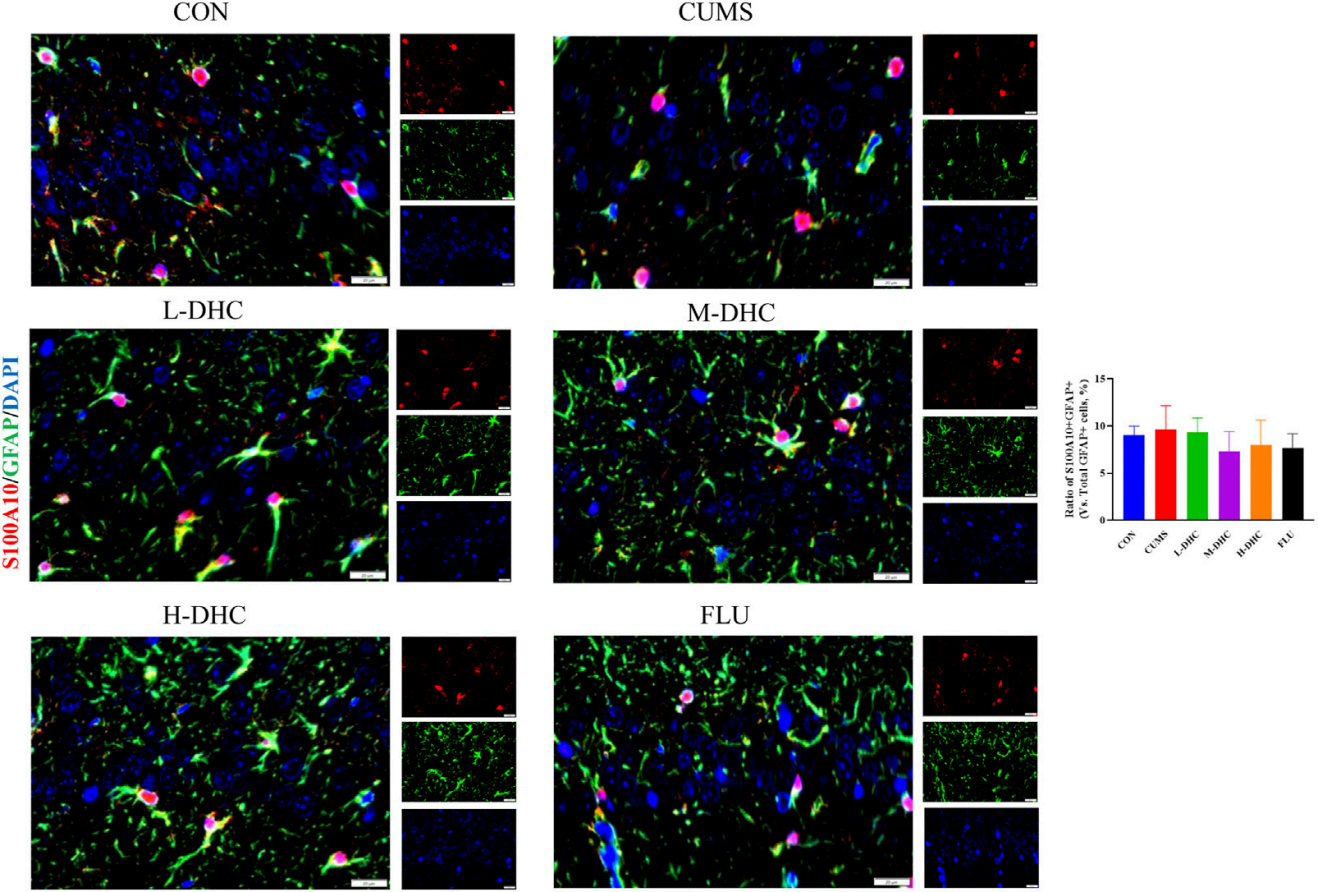
Effects of DHC or fluoxetine on A2 astrocyte activation in CUMS-induced depression in mice. Red fluorescence represented S100A10 positive cells, and green fluorescence represented GFAP positive cells.

**FIGURE 8:**
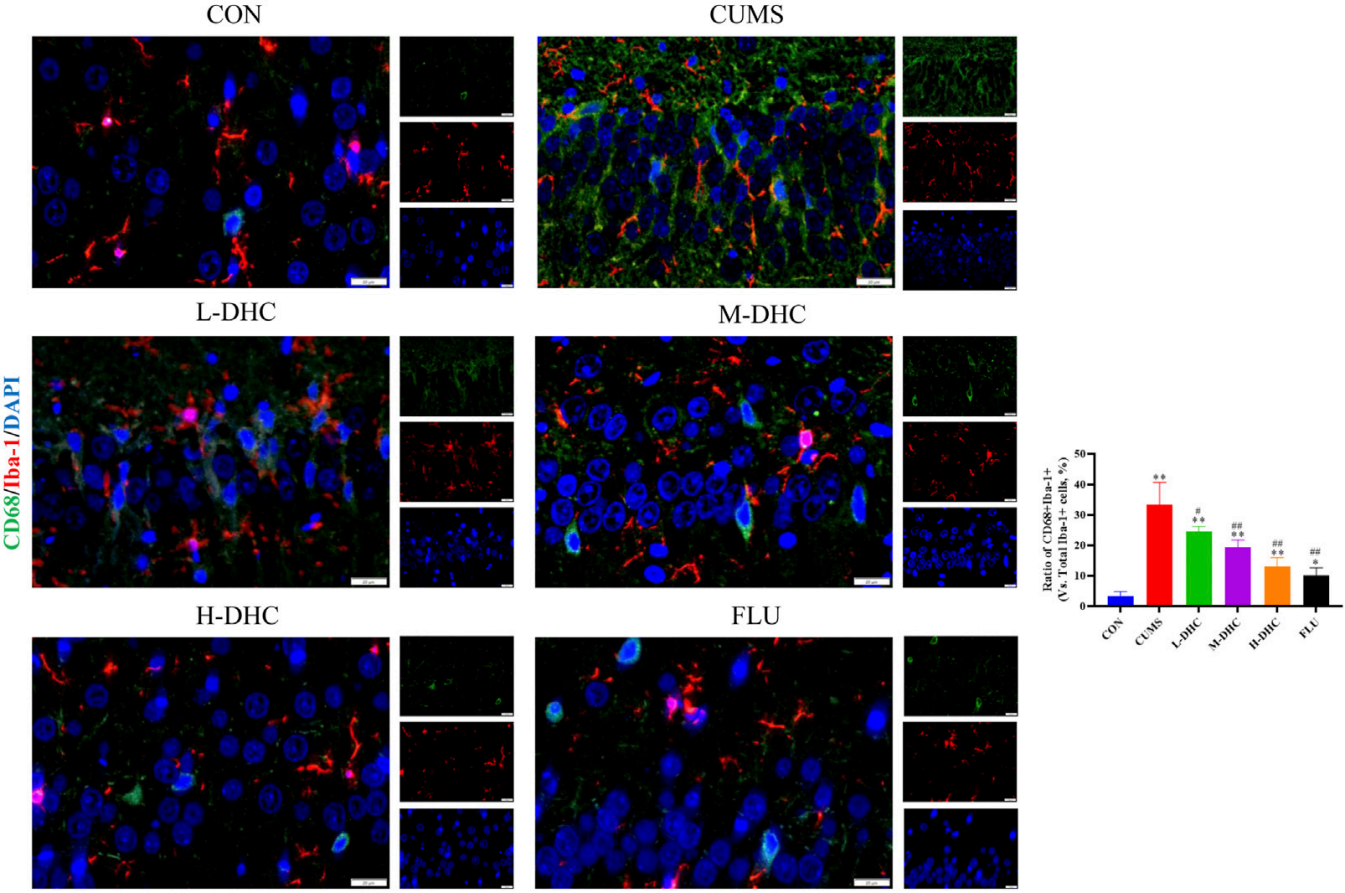
Effects of DHC or fluoxetine on microglia activation in CUMS-induced depression in mice. Red fluorescence represented Iba-1 positive cells, and green fluorescence represented CD68 positive cells. ^**^ *p* < 0.01, compared with the control (CON) group; ^##^ *p* < 0.01, compared with the CUMS group.

Since the microglia and astrocytes are the main glial cells that mediate the inflammatory response of the central nervous system, we detected the activation of A1/A2 astrocytes and microglia by immunofluorescence assay. As shown in Figure 6, the representative A1 astrocyte marker C3 and the number of GFAP fluorescence positive co-marker cells in the hippocampus of mice in the DHC and FLU treatment groups were significantly decreased compared with the CUMS group. There was no significant change in the number of A2 astrocyte marker S100A10 cells among the groups (Figure 7). The results in Figure 8 show that the number of representative microglia markers CD68 and Iba-1 fluorescence positive co-marker cells in the hippocampus of mice in the DHC and FLU treatment groups were significantly decreased compared with that in the CUMS group. These results suggested that A1 astrocytes and microglia were activated in CUMS mice, and DHC intervention could inhibit their activation, thereby alleviating depression.

### Activated microglia induced activation of A1 astrocytes

**FIGURE 9:**
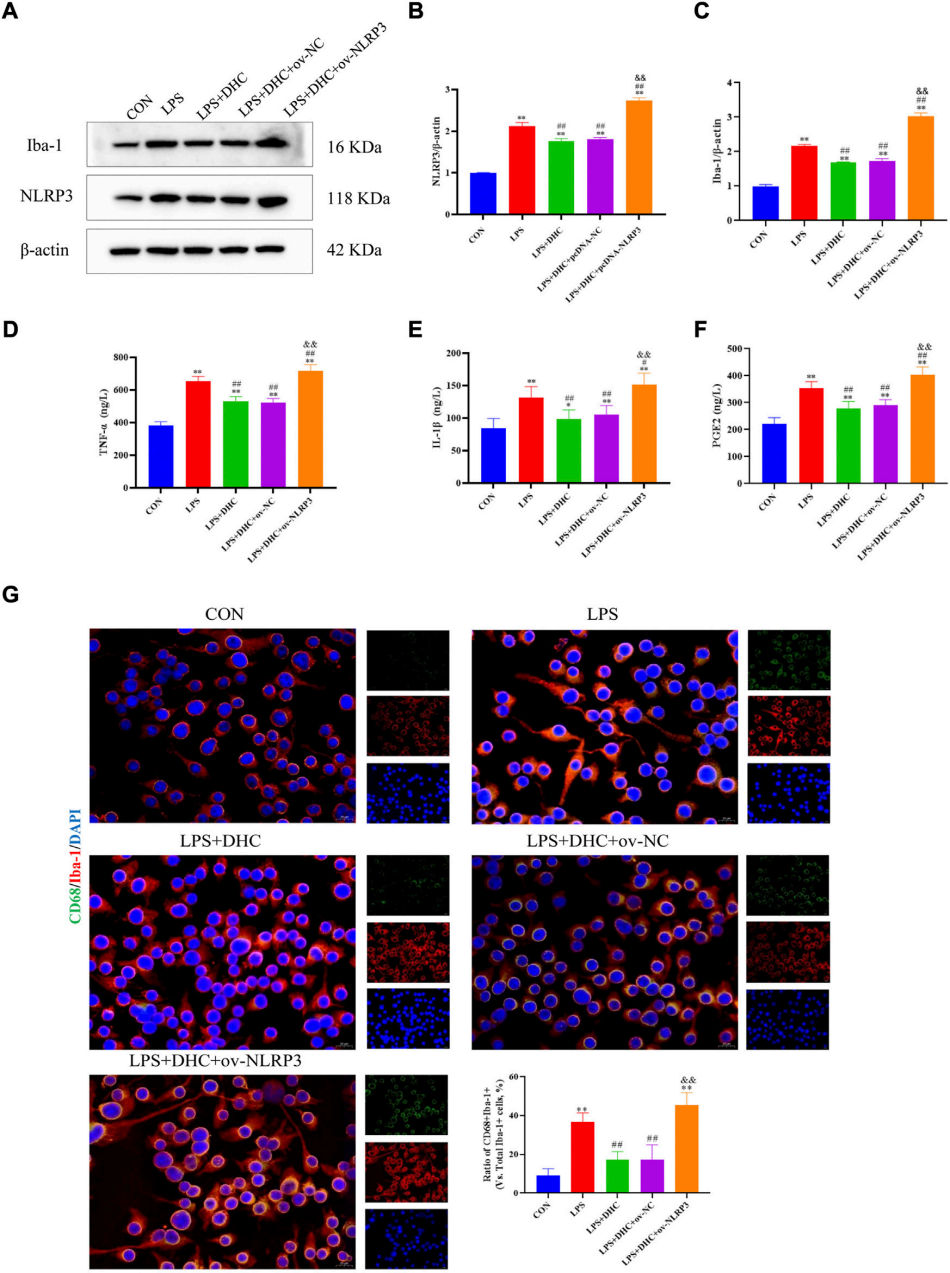
Effects of DHC on LPS-induced activation of microglia. BV2 cells were incubated with DHC (100 mg/L) and 100 ng/ml LPS for 12 h ov-NC or ov-NLRP3 plasmid was transfected into BV2 cells 6 h before LPS induction. **(A)** The expression levels of NLRP3 and Iba-1 protein were determined by western blotting; **(B)** the relative intensity of NLRP3 protein was shown as a bar graph; **(C)** the relative intensity of Iba-1 protein was shown as a bar graph; **(D)** concentration of TNF-α in each group; **(E)** concentration of IL-1α in each group; **(F)** concentration of PGE2 in each group; and **(G)** the microglia was subjected to immunofluorescent staining with CD68 (green), Iba-1 (red), and DAPI (blue). ^*^ *p* < 0.05 and ^**^ *p* < 0.01, compared with the control (CON) group; ^#^ *p* < 0.05 and ^##^ *p* < 0.01, compared with the LPS group; ^&&^ *p* < 0.01, compared with the LPS + DHC group.

**FIGURE 10:**
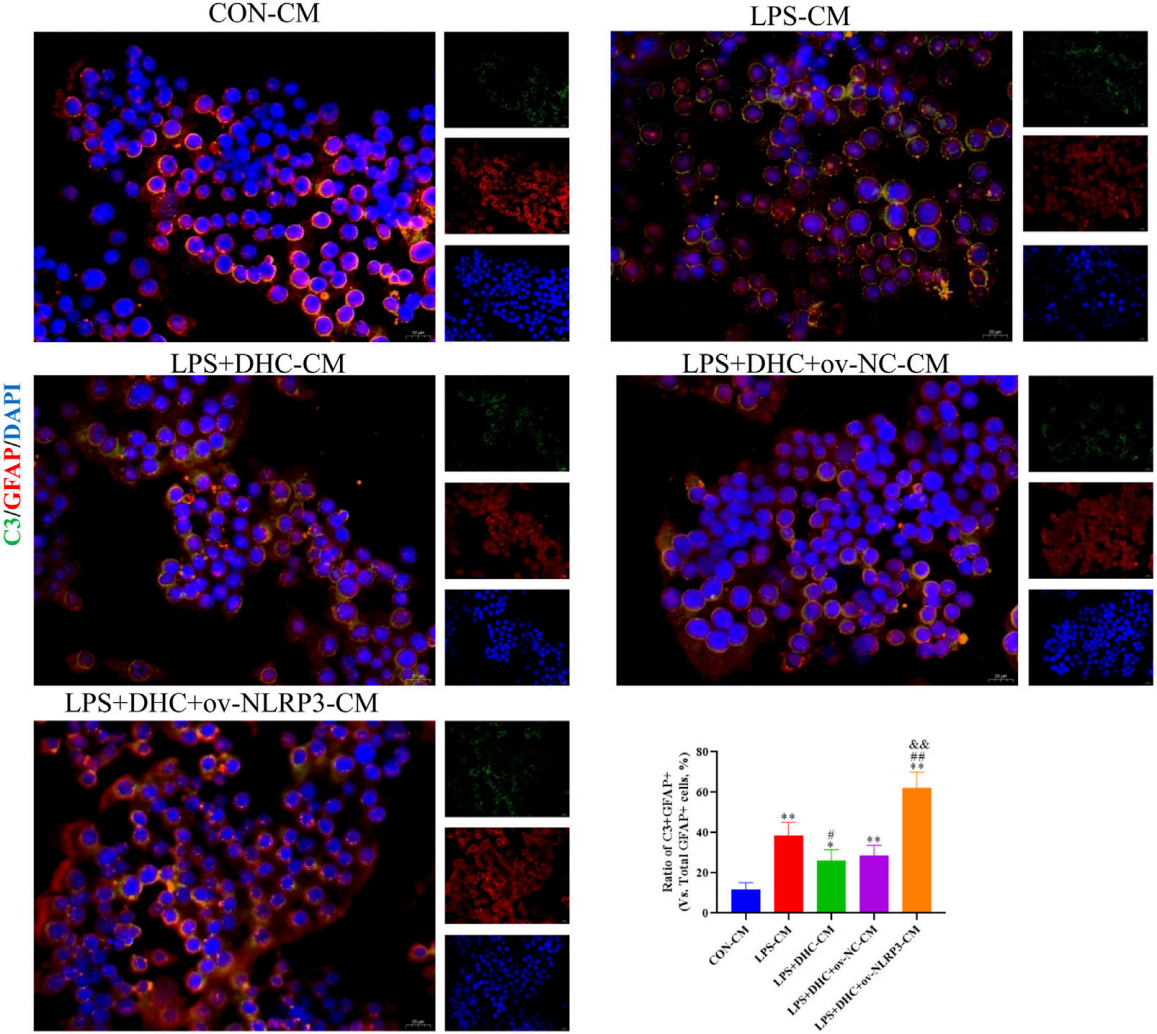
Effects of the microglia-conditioned medium on astrocyte activation. BV2 cells were incubated with DHC (100 mg/L) and 100 ng/ml LPS for 12 h ov-NC or ov-NLRP3 plasmid was transfected into BV2 cells 6 h before LPS induction. Conditioned media of microglia were collected from each group, followed by intervention of astrocytes for 24 h. The astrocytes were subjected to immunofluorescent staining with C3 (green), GFAP (red), and DAPI (blue). ^*^ *p* < 0.05 and ^**^ *p* < 0.01, compared with the control (CON) group; ^##^ *p* < 0.01, compared with the LPS group; ^&&^ *p* < 0.01, compared with the LPS + DHC group.

**FIGURE 11:**
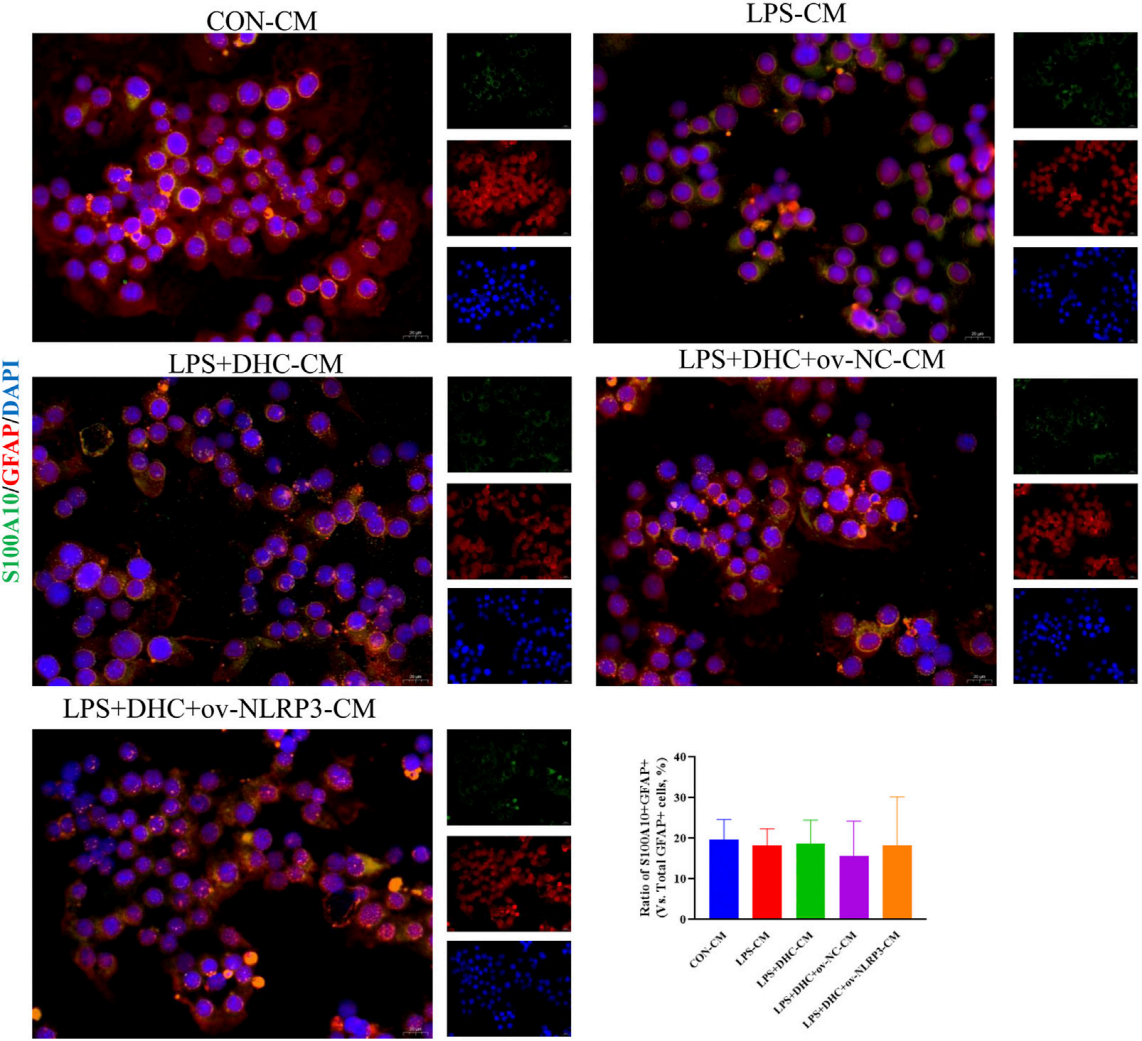
Effects of microglia-conditioned medium on astrocyte activation. BV2 cells were incubated with DHC (100 mg/L) and 100 ng/ml LPS for 12 h ov-NC or ov-NLRP3 plasmid was transfected into BV2 cells 6 h before LPS induction. Conditioned media of microglia were collected from each group, followed by intervention of astrocytes for 24 h. The astrocytes were subjected to immunofluorescent staining with S100A10 (green), GFAP (red), and DAPI (blue).

To further clarify the regulatory role of microglia NLRP3 in the activation of astrocyte, we first studied the LPS-induced effects on microglia activation, and clearly DHC reversed the effect on this process. As shown in Figure 9, compared with the control group, the expression levels of NLRP3, Iba-1, TNF-α, IL-1β, and PGE2 and the positive co-marker number of representative microglial markers CD68 and Iba-1 in the LPS-induced group were significantly increased. DHC intervention reduced NLRP3-associated inflammasome protein expression, inflammatory factors concentrations, and CD68/Iba-1 positive co-marker numbers, while NLRP3 overexpression significantly reversed the DHC effect. These results suggested that DHC could inhibit LPS-induced microglial activation, while NLRP3 overexpression could significantly reverse the effect of DHC. Astrocytes were further cultured in a microglia-conditioned medium to observe the activation. As shown in Figure 10, compared with the CON-CM group, LPS-CM stimulation significantly upregulated the positive co-marker number of C3 and GFAP, representative A1 markers of astrocytes; LPS + DHC-CM intervention could reduce the positive co-marker number of C3/GFAP, while NLRP3 overexpression could significantly inhibit the effect of DHC. However, the microglia-conditioned medium had no effect on A2 astrocyte activation (Figure 11). These results suggested that the NLRP3 overexpression of microglia could promote the activation of A1 astrocytes.

## Discussion

Depression is a serious emotional disorder characterized by anhedonia, loss of energy, loss of appetite, cognitive impairment, motor retardation, and suicidal tendencies (Pitsillou et al., 2020). The main pathological features of depression include significantly reduced volume and weight of the cortex and hippocampus, significantly reduced number and density of astrocytes, and extensive neuroinflammation (Rajkowska and Stockmeier, 2013). Depression is a disorder caused by genetic factors, environmental factors, and individual factors, and its pathogenesis is complex, among which inflammation is one of the main pathogenic mechanisms (Gaiteri et al., 2014). Inflammasome, especially NLRP3 inflammasome, a member of the NLR family, is a major player in mediating neuroinflammation (Huang et al., 2021). The NLRP3 inflammasome also mediates the transformation of microglia from resting to activated morphology, and activated microglia is an important participant in the neuroinflammatory response in the hippocampus of stress-model mice (Wu et al., 2021). It was found that microglia were activated and transformed into proinflammatory phenotype, releasing proinflammatory cytokines and affecting astrocyte activity under chronic stress (Borggrewe et al., 2020). Therefore, the purpose of this study was to investigate the role of DHC on microglia activation and astrocyte A1/A2 phenotype in depression and the role of NLRP3 inflammasome in these effects. The results revealed that DHC could inhibit the NLRP3 inflammasome-mediated microglia activation; inhibit the secretion and release of TNF-α, IL-1α, and PGE2; and then inhibit the activation of A1 astrocytes, thus alleviating depression-like behavior in mice.

Initially, we performed behavioral tests and found that the CUMS mice had an obvious depressive-like behavior. Following the administration of DHC, the sucrose intake of CUMS mice was significantly increased, and immobile time in FST and TST of CUMS mice was significantly reduced. Concerning H&E and Nissl staining assay, it revealed that the hippocampal neurons were abundant and neatly arranged in bands in CUMS mice following DHC administration. Abnormal levels of brain neurotransmitters such as 5-HT and DA are closely related to the formation of depression. This study revealed that the concentrations of 5-HT and DA in the serum and hippocampus were significantly increased in CUMS mice following DHC administration. The aforementioned data indicated that DHC could ameliorate depressive-like behaviors, attenuate neuron damage, and increase neurotransmitter concentration.

Dehydrocorydaline is an effective extract from the tuber of *Corydalis yanhusuo* (Y.H.Chou and Chun C. Hsu) W.T. Wang ex Z.Y. Su and C.Y. Wu (*Papaveraceae*; *Corydalis rhizoma*) and is often used in the treatment of cardiovascular diseases. A previous study reports that dehydrocorydaline can exert antidepressant effects by inhibiting monoamine transporter uptake in depressed CUMS-mouse models (Jin et al., 2019), while its anti-neuroinflammatory effects have not been reported. Neuroinflammatory response is the main manifestation of almost all central nervous system disorders (Elbert et al., 2022), including acute trauma, infection, neurodegenerative disorders, and psychiatric disorders such as depression. This study revealed that DHC could decrease the proinflammatory factor levels of the serum and hippocampus in CUMS mice, indicating that DHC might alleviate depression by anti-neuroinflammation.

Microglia and astrocytes play a central role in mediating the initiation and amplification of neuroinflammatory responses in neuroimmune cells (Kane and Drew, 2021; Villarreal et al., 2021). Microglia are the first cell population to detect and respond to CNS homeostasis disorder by secreting inflammatory cytokines and chemokines during CNS injury, and astrocytes are then successively activated and transformed into reactive astrocytes by these signals (Villarreal et al., 2021). The cross-signaling between microglia and astrocytes is crucial to determine the intensity and timing of this glial cascade (Matejuk and Ransohoff, 2020). Inflammasome is the main responder of neuroinflammatory response mediated by glial cells in various injury modes, and NLRP3 inflammasome has become a research hotspot due to its high expression and wide distribution (Li et al., 2022; Zhou et al., 2022). In this study, we observed that DHC significantly inhibited the activation of NLRP3 inflammasome-associated signaling pathways, microglia, and A1 astrocytes in the CUMS mice. The aforementioned results indicated that DHC might play an antidepressant role by inhibiting the activation of microglia and their downstream NLRP3 inflammasome, thereby inhibiting the activation of A1 astrocytes. However, the relationship between microglia and astrocyte and neuron crosstalk needs to be further studied.

Studies have shown that TNF-α, IL-1α, and PGE2 secreted by activated microglia under inflammatory stimulation are necessary for inducing the activation of A1 astrocytes (Nishimoto et al., 2021; Gallo et al., 2022). In this study, LPS-activated microglia were first observed, and NLRP3 overexpression promoted the activation of microglia, suggesting that NLRP3 may mediate the activation of microglia, and DHC has a significant reversal effect on this process. Furthermore, we incubated astrocytes with a conditioned medium of microglia, and it was found that A1 astrocytes were activated by the conditioned medium containing LPS or ov-NLRP3, indicating that activated microglia promoted the secretion of TNF-α and other inflammatory mediators through the NLRP3 inflammasome pathway and then induced the activation of A1 astrocytes.

## Conclusion

In summary, the results of this study found that the microglia, astrocytes, and NLRP3 inflammasome pathways were activated during the occurrence and development of depression, and DHC had a significant inhibitory effect on their activation. On this basis, the effect of NLRP3 overexpression on the activation of microglia and the effect of the microglia-conditioned medium on the activation of A1 astrocytes were studied *in vitro*. These results suggest that DHC may play an antidepressant role by inhibiting microglia–NLRP3 inflammasome pathway activation–A1 astrocyte activation. However, there are still several limitations in our study. First, the effect of knocking down or overexpressing NLRP3 on the release of IL-18 and IL-1 β is unclear and will be explored in our next study. Second, the specific mechanism of NLRP3 inflammatory bodies in depression needs to be studied, which will also be discussed in the future. The results of this study are expected to provide a theoretical basis for the screening of therapeutic targets for DHC.

## Data Availability

The raw data supporting the conclusions of this article will be made available by the authors, without undue reservation.
